# *LILR* genotype imputation with attribute bagging (LIBAG): leukocyte immunoglobulin-like receptor copy number imputation system

**DOI:** 10.3389/fimmu.2025.1559301

**Published:** 2025-04-07

**Authors:** Seik-Soon Khor, Kouyuki Hirayasu, Yosuke Kawai, Hie Lim Kim, Masao Nagasaki, Katsushi Tokunaga

**Affiliations:** ^1^ Genome Medical Science Project, National Center for Global Health and Medicine, Tokyo, Japan; ^2^ Singapore Centre for Environmental Life Sciences Engineering, Nanyang Technological University, Singapore, Singapore; ^3^ Advanced Preventive Medical Sciences Research Center, Kanazawa University, Kanazawa, Ishikawa, Japan; ^4^ Department of Immunology, Graduate School of Medical Sciences, Kanazawa University, Kanazawa, Ishikawa, Japan; ^5^ Asian School of the Environment, Nanyang Technological University, Singapore, Singapore; ^6^ Division of Biomedical Information Analysis, Medical Research Center for High Depth Omics, Medical Institute of Bioregulation, Kyushu University, Fukuoka, Fukuoka, Japan; ^7^ Center for Genomic Medicine, Graduate School of Medicine, Kyoto University, Kyoto, Kyoto, Japan

**Keywords:** LILR, LIBAG, imputation, copy number, SNP, GWAS, CNV, HLA

## Abstract

There are ten leukocyte immunoglobulin (Ig)-like receptor (*LILR*) genes, i.e., five genes encoding activating receptors (*LILRA1, LILRA2, LILRA4, LILRA5*, and *LILRA6*) characterized by their truncated cytoplasmic tails, and five genes encoding inhibitory receptors (*LILRB1, LILRB2, LILRB3, LILRB4*, and *LILRB5*) characterized by their extended cytoplasmic tails containing immunoreceptor tyrosine-based inhibitory motifs (ITIMs). Among these, *LILRB3*, *LILRA6*, and *LILRA3* are known for harboring high frequencies of copy number variations (CNVs). However, the presence of CNVs in the leukocyte receptor complex (LRC) region complicates single nucleotide polymorphism (SNP) association analysis within commercially available SNP microarray datasets. This study introduces LILR Genotype Imputation with Attribute Bagging (LIBAG), a novel method for determining CNVs in *LILRB3, LILRA6*, and *LILRA3* from commercially available SNP genotyping array datasets. *LILRA6* CNV imputation accuracy peaked at 98.0% for the Infinium Japanese Screening Array, followed by 97.4% for Axiom Japonica V2, 97.3% for Axiom Japonica Array NEO, and 94.3% for Axiom Japonica V1, with the lowest recorded accuracy of 93.6% for the Axiom Genome-wide ASI1 array. For the 1000 Genomes Project (1kGP) dataset, *LILRA6* CNV imputation achieved peak accuracies of 94.5% for 1kGP-EAS (East Asian), 86.6% for 1kGP-AMR (Admixed American), 83.8% for 1kGP-EUR European), and 75.0% for 1kGP-AFR (African), particularly after the 20 kb flanking region. Similarly, imputation accuracy for *LILRA3* CNV progressively increased, peaking at the 80 kb flanking region. Accuracy reached 1kGP-AMR, reaching 99.2% and 98.9% for 1kGP-AFR, 98.7% for 1kGP-EUR, and 97.5% for 1kGP-EAS. Investigating the *LILR* copy number (CN) in diseases associated with HLA class I molecules will provide further insights into disease pathogenesis.

## Introduction

**Figure 1:**
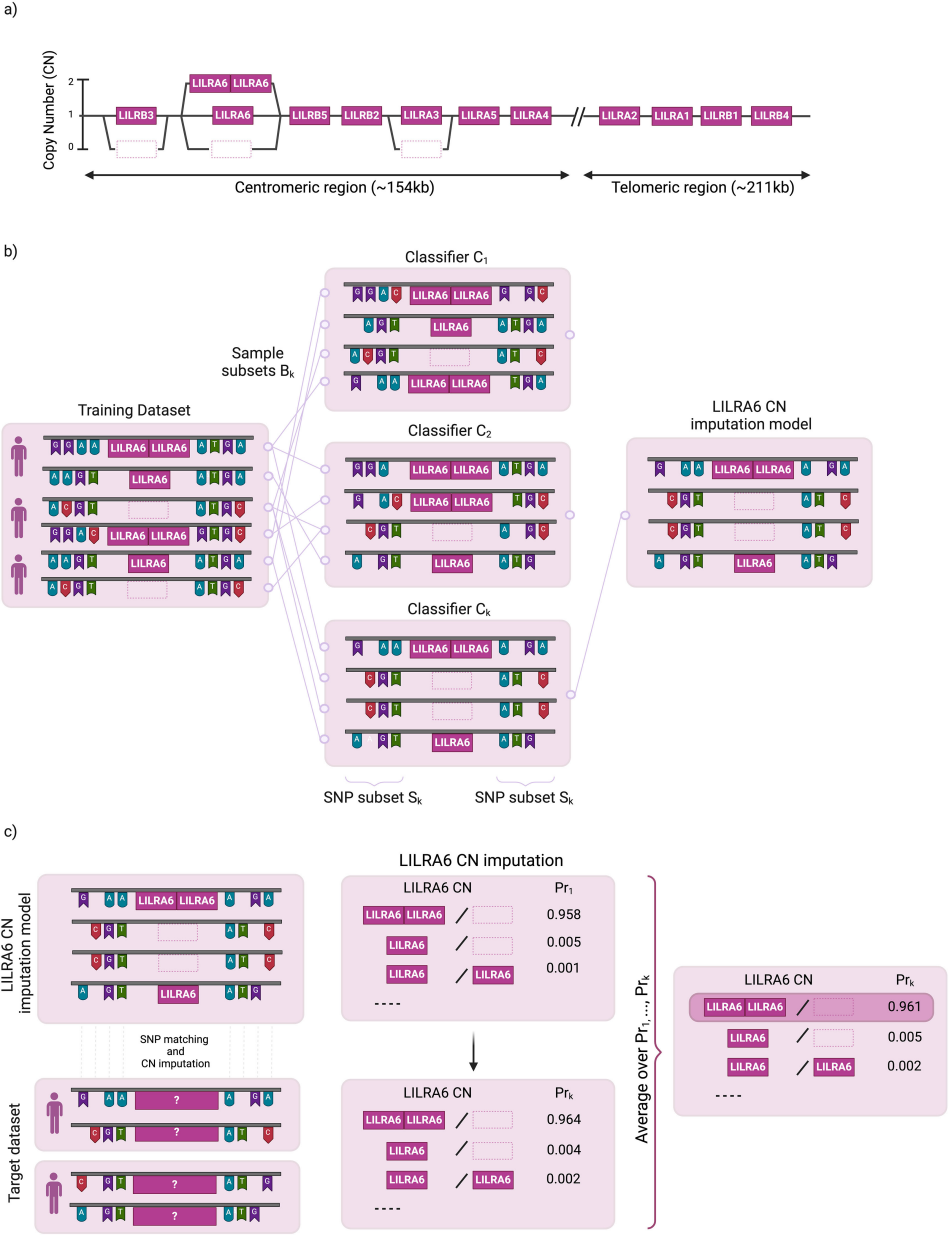
Schematic overview of LILR copy number (CN) imputation. **(a)** Copy Number Distribution of 11 LILR Genes **(b)** Overview of the LIBAG prediction algorithm. LIBAG constructs classifiers by taking K bootstrap samples from a reference set of individuals with known LILR CN and SNP genotypes. Each sample, Bk, includes some individuals multiple times, leaving approximately 37% as out-of-bag. For each Bk, a classifier Ck is trained using an optimal SNP subset Sk. **(c)** SNPs from the target dataset are aligned with those in the LILR CN model to estimate LILR CN for all K classifiers LILR CN with the highest aggregated probabilities (Pr_k_) is determined as the final predicted LILR CN.

The leukocyte immunoglobulin (lg)-like receptor (*LILR*) gene family (also known as immunoglobulin-like transcript (ILT)) or LIR family) is mapped to the leukocyte receptor complex (LRC) region on chromosome 19q13.4. This region contains multigene families of the innate immune system belonging to the lg superfamily, such as killer lg-like receptors (KIR), leukocyte-associated lg-like receptors (LAIRs), natural cytotoxicity receptor 1 (NCR1) and the Fc-alpha receptor (FcAR) (1, 2). LILRs are classified into two major groups, i.e., LILRA and LILRB. LILRAs (**Figure 1**) consist of five activating receptors (LILRA1, LILRA2, LILRA4, LILRA5, and LILRA6), characterized by truncated cytoplasmic tails and interaction with the γ-chain of FcεRI through a charged arginine residue in the transmembrane domain. This interaction facilitates transduction of the activating signal via an immunoreceptor tyrosine-based activation motif (ITAM) (3). In contrast, LILRBs comprise five inhibitory receptors (LILRB1, LILRB2, LILRB3, LILRB4, and LILRB5), characterized by long cytoplasmic tail with immunoreceptor tyrosine-based inhibitory motifs (ITIM). LILRA3 is a soluble protein lacking both transmembrane and cytoplasmic domains (4, 5).

Immune cell types including myeloid and lymphoid lineages broadly express *LILR* genes. In contrast with KIRs, which interact with polymorphic epitopes on the α1 and α2 domains of HLA class I members, LILRs bind to conserved motif epitopes on the α3 domain and/or with the highly conserved β2-microglobulin structure (6). At the single-gene level, all four LILR receptors (LILRA1, LILRA3, LILRB1, and LILRB2) exhibit strong affinity for HLA class I molecules, especially LILRB1 and LIRB2 (7–9).

Similar to the neighboring *KIR* genes*, LILR* genes exhibit high levels of copy number variation (CNV) and allelic variations (9, 10); *LILRA6* shows a CNV ranging from 0 to 4 copies per haplotype, a result of nonallelic homologous recombination between *LILRB3* and *LILRA6* (9, 11, 12). In addition, 0–2 copies of *LILRA3* have been detected in 48 human cell lines sourced from the International Histocompatibility Working Group (12). LILRA3 with a deficiency in the extracellular domain (13) and LILRA3 with a premature termination codon have also been reported (14, 15). However, experimental CNV determination of *LILRA6* and *LILRA3* remains laborious because of the homology (98%) between *LILRB3* and *LILRA6*. In this paper, we propose a novel method to determine *LILR* CNV from whole-genome sequencing (WGS) datasets and a novel SNP-based imputation method to determine the CNV of *LILR*s that show CNV (*LILRB3, LILRA6*, and *LILRA3*). This project is part of the 19th International HLA & Immunogenetics Workshop (https://ihiw19.org/), focusing on the “Leukocyte Receptor Complex (LRC) Structure and Polymorphism.”

## Materials and methods

### Samples

#### Japanese dataset

A total of 418 Tokyo Healthy Control (THC) samples were collected from healthy Japanese individuals in Tokyo, Japan; 182 Japanese individuals from a replication cohort were recruited from the National Hospital Organization Nagasaki Medical Center (NMC), Nagasaki, Japan. Healthy control participants were confirmed to have no significant disease at sample collection. All the healthy individuals This study was approved by the Ethical Committee of the National Center for Global Health and Medicine and National Hospital Organization Nagasaki Medical Center. Written informed consent was obtained from all participants.

#### 1000 genomes project dataset

Short-read whole-genome sequencing (srWGS) 175 1kGP-AFR (African), 268 1kGP-AMR (Admixed American), 140 1kGP-EAS (East Asian) and 100 1kGP-EUR (European) were downloaded from the International Genome Sample Resource database (https://www.internationalgenome.org/data-portal/data-collection/30x-grch38). The SNP microarray dataset, genotyped using the Illumina Omni2.5 microarray (Illumina, CA, US), was downloaded from http://ftp.1000genomes.ebi.ac.uk/vol1/ftp/release/20130502/supporting/hd_genotype_chip/.

#### SNP genotyping

Genome-wide SNP genotyping was performed on 418 Japanese THC samples using the following arrays: Axiom Genome-wide ASI1 array (Thermo Fisher Scientific, MA, US), Axiom Japonica V1 (Thermo Fisher Scientific, MA, US), Axiom Japonica V2 (Thermo Fisher Scientific, MA, US), Axiom Japonica Array NEO (Thermo Fisher Scientific, MA, US) and Infinium Japanese Screening Array (Illumina, CA, US). CEL format output files were generated according to the manufacturer’s recommended procedure. Genotyping of each variant was performed using Apt software (ver. 2.10.2.2). All samples met quality control criteria as defined by the manufacturer’s recommended workflows. Probe intensity clustering results were classified using SNPolisher. Quality controls for SNPs involved removing autosomal SNPs with a frequency of ≤ 5%, a call ≤ 95%, and deviation from Hardy–Weinberg equilibrium (*p* < 1e-5).

#### CNV calling from PacBio sequencing dataset

CNV of *LILRB3, LILRA6*, and *LILRA3* was determined from the short-read whole-genome sequencing (srWGS) dataset for both the Japanese and 1kGP sample sets using the JoGo-*LILR* CNV Caller (16) (https://jogo.csml.org/JoGo-LILR/). Briefly, the JoGo-*LILR* CN Caller (16) employs three sequential steps in determining the CN: mapping whole-genome sequencing reads to GRCh38 with decoy sequences (GRCh38DH); calculating normalized read depths for *LILRB3, LILRA6*, and *LILRA3* using CNVNator (17); and calling of CN types and estimating CN haplotypes based on the *LILRB3, LILRA6, and LILRA3* cluster plots generated in step 2. Internal validation of the results was performed using the trio datasets from the 1kGP data.

#### LIBAG – *LILR* genotype imputation with attribute bagging

LIBAG is a novel method for determining CNV in *LILRB3, LILRA6*, and *LILRA3* from commercially available SNP genotyping array datasets (**Figure 1**). This method combines attribute bagging, an ensemble classifier method, with haplotype inference for SNPs and *LILR* CNV. Attribute bagging improves the accuracy and stability of imputation references through bootstrap aggregation and random variable selection (18).

LIBAG constructs classifiers by taking K bootstrap samples from a reference set of individuals with known LILR CN and SNP genotypes. Each sample, B_k_, includes some individuals multiple times, leaving approximately 37% as out-of-bag. For each B_k_, a classifier C_k_ is trained using an optimal SNP subset S_k_ (**Figure 1**). LIBAG was used to select a random subset of SNPs (S_k_) flanking *LILRB3, LILRA6*, and *LILRA3* for each bootstrapped sample. This subset was optimized by iteratively adding SNPs until maximum imputation accuracy is achieved, based on out-of-bag samples (samples that are not included in the current bootstrapped samples). The conditional probability of each possible *LILR* CN was calculated using estimated haplotype frequencies for the selected SNP subset, allowing the classifier to predict the *LILR* CN by maximizing posterior probabilities. Subsequently, an ensemble prediction was generated by averaging the posterior probabilities across all the individual classifiers. This method avoids overfitting and improves the CN prediction stability. To predict the CN for *LILR* genes of a new individual, LIBAG combines the probabilities from all classifiers and selects the *LILR* CN with the highest average probability.

Simulation studies on the flanking SNPs region, ranging from 10 to 100kb, were conducted to maximize *LILR* CNV imputation accuracy while minimizing computer power consumption. A call threshold (CT) was generated for each imputed *LILR* CNV pair, representing the confidence of the imputation. CT cutoff evaluations were performed to determine the delicate balance between call rate and imputation accuracy.

After constructing the LILR CN imputation model (**Figure 1**), the SNPs in the target dataset are matched to the SNPs set in our LILR CN imputation model to impute all possible LILR CN for all K classifiers. We leverage the aggregation of the K predictors to maximize imputation accuracy; LILR CN with the highest aggregated probabilities (Pr) is determined as the final predicted LILR CN.

#### Validation of *LILR* imputation reference

External validation of the generated *LILR* gene imputation references was performed by comparing the *LILR* CNV calling results with the *LILR* CNV imputation results in the 182 NMC Japanese dataset.

## Results

LIBAG, an imputation toolkit developed as an integral component of the pre-existing HIBAG R statistics package (18, 19), facilitates CNV imputation within *LILRB3, LILRA6*, and *LILRA3*. This CNV imputation process is compatible with various commercially available SNP genotyping array datasets, including those from Thermo Fisher Scientific (MA, USA) and Illumina (CA, USA). In order to facilitate a high-quality LILR CNV imputation system for the datasets from these two companies (**Figure 1**), we have built a high-quality *LILR* CNV imputation system consisting of SNP data from genome-wide SNP microarray genotyping and LILR CNV genotyping data generated using the JoGo-*LILR* CN Caller. Once the *LILR* CNV imputation system is built, imputation can be performed by matching SNPs between the *LILR* CNV imputation model and the target dataset. Call thresholds, representing the confidence of *LILR* CNV imputation quality, were assigned to each imputed *LILR* CNV haplotype set.

### *LILR* CNV distributions

**Figure 2:**
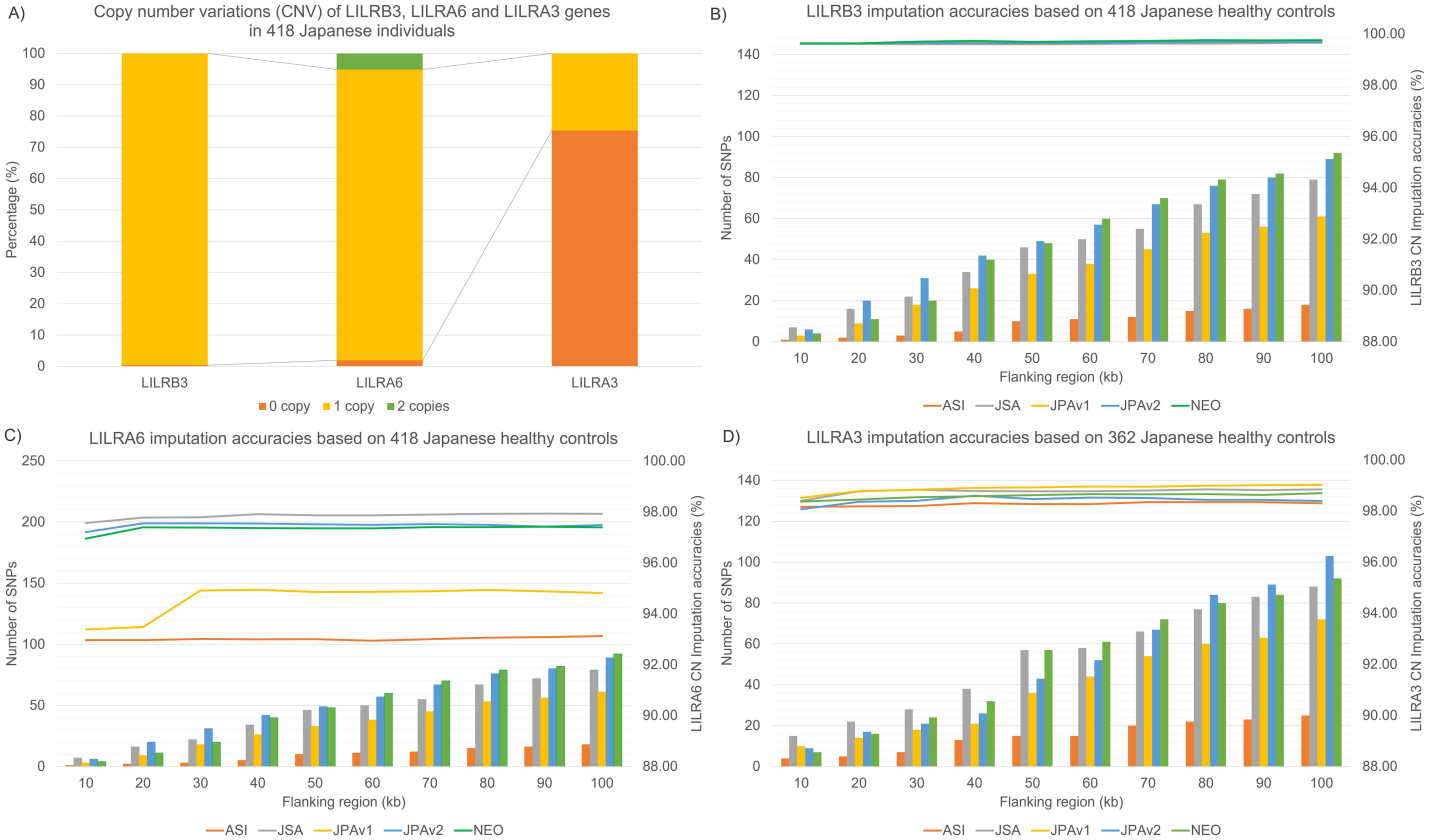
CN distributions of *LILR*s in the Japanese population and CN imputation accuracies across five Asian/Japanese-specific SNP microarray platforms. **(A)** CN distribution of *LILRB3, LILRA6, and LILRA3* in 418 Japanese individuals. **(B)** Internal validations of *LILRB3* CN imputation accuracies across five SNP microarray platforms, including the number of SNPs used for model building in flanking regions spanning from 10–100 kb around the *LILRB3* gene **(C)** Internal validations of *LILRA6* CN imputation accuracies across five SNP microarray platforms and the number of SNPs used for model building in flanking regions spanning from 10–100 kb around the *LILRA6* gene **(D)** Internal validations of LILRA3 CN imputation accuracies across five SNP microarray platforms, including the number of SNPs used for model building in flanking regions spanning from 10–100 kb around the *LILRA3* gene.

The JoGo-*LILR* CN Caller was used to call the *LILR* CNV for *LILRB3, LILRA6, and LILRA3* from the WGS dataset, comprising Japanese and 1kGP datasets. In the Japanese dataset (**Figure 2A**), *LILRB3, LILRA6, and LILRA3* CNVs exhibited uneven distributions. *LILRA6* showed the most CNV polymorphism (92.6% 1CN, 5.1% 2CN, 1.9% 0CN), followed by *LILRA3* (75% 0CN, 24% 1CN). *LILR*B3 displayed the least CNV polymorphism (0.3% of 0CN, 99.6% of 1CN).

**Figure 3:**
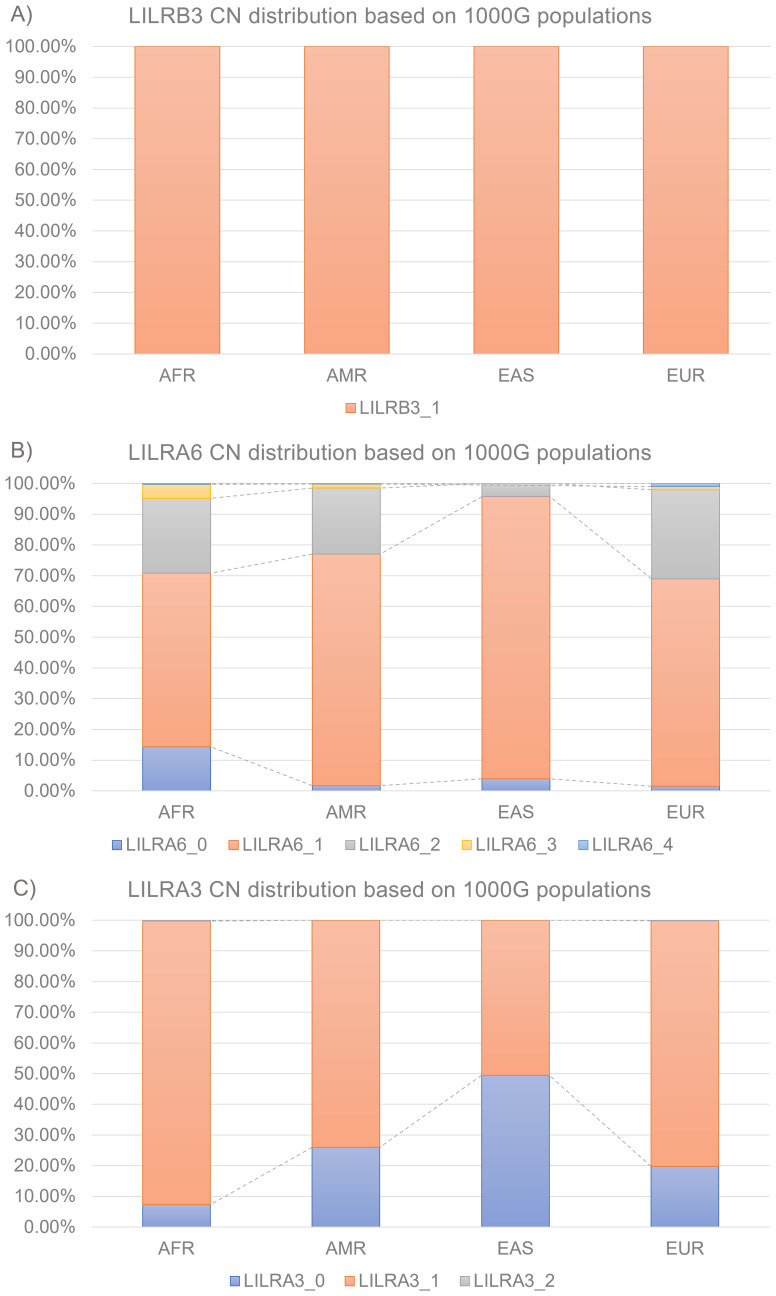
Distributions of *LILR* CN across the 1000 Genome Project super-populations **(A)** *LILRB3* **(B)** *LILRA6* **(C)** *LILRA3*. AFR, African; AMR, Admixed American; EAS, East Asian; EUR, European; CN, Copy Number.

Similarly, *LILRA6* CN exhibited the most polymorphism in the 1kGP dataset (**Figure 3B**). 1kGP-AFR showed five CN types: 56.6% 1CN, 24.3% 2CN, 14.3% 0CN, 4.6% 3CN, and 0.3% 4CN. Both 1kGP-AMR and 1kGP-EUR showed five CN types: 75.4% 1CN, 21.5% 2CN, 1.7% 0CN, 1.3% 3CN, and 0.2% 4CN; 67.5% 1CN, 29.0% 2CN, 1.5% 0CN, 1.0% 3CN, and 1.0% 4CN. Finally, 1kGP-EAS showed only three CN types: 91.8% 1CN, 4.3% 2CN, 3.9% 0CN.

*LILRA3* was the second most polymorphic *LILR* identified in this study (**Figure 3C**). Both 1kGP-AFR and 1kGP-EUR showed three CN types: 92.5% 1CN, 7.3% 0CN, and 0.3% 2CN; and 80.1% 1CN, 19.8% 0CN, and 0.2% 2CN. However, 1kGP-AMR and 1kGP-EAS showed only two CN types: 74.0% 1CN, 26.0% 0CN, 50.5% 1CN, and 49.5% 0CN.

Finally, *LILRB3* showed no CNV in the 1kGP dataset with all samples carrying 1CN (**Figure 3A**).

### Japanese *LILR* CNV imputation accuracies

Simulation studies were performed on the SNP region flanking 10–100 kb from the center of *LILRB3, LILRA6*, and *LILRA3*. The objective was to improve the *LILR* CNV imputation accuracy while reducing computer power usage. The number of SNPs used for *LILR* imputation varied based on the evaluated SNP genotyping array. For instance, (**Figures 2B–D**) the Axiom Genome-wide ASI1 array, an earlier SNP genotyping version, contained the fewest usable SNPs, ranging from 1 to 18 SNPs. In contrast, Japanese-specific SNP genotyping arrays such as the Infinium Japanese Screening Array with 7-79 SNPs, Axiom Japonica V1 with 3-61 SNPs, Axiom Japonica V2 with 6-89 SNPs, and Axiom Japonica Array NEO with 4-92 SNPs, exhibit a wide range of usable SNPs for *LILR* CNV imputation.

Internal validations assessed *LILR* CNV imputation accuracies (**Figures 2B–D**) over an SNP region extending 10–100 kb from the center of the *LILRB3, LILRA6*, and *LILRA3* genes. We found a clear relationship between the number of SNPs utilized in the *LILR* imputation model and the imputation accuracies; for example, the CNV imputation accuracies for *LILRA6* (**Figure 2C**) and *LILRA3* (**Figure 2D**) increased notably from the 20 kb to the 30 kb flanking region before plateauing beyond 30 kb. *LILR* CNV imputation accuracy also depends heavily on SNPs’ availability across various SNP genotyping arrays. Specifically, the *LILRA6* CNV imputation accuracy peaked at 98.0% for the Infinium Japanese Screening Array, followed by 97.4% for Axiom Japonica V2, 97.3% for Axiom Japonica Array NEO, and 94.3% for Axiom Japonica V1, with the lowest recorded accuracy of 93.6% for the Axiom Genome-wide ASI1 array. In contrast, *LILRA3* and *LILRB3* CNV imputation accuracies demonstrated minimal variation (98.4-99.0% and 99.7-99.8%, respectively) across the five SNP genotyping arrays. A Japanese dataset (182 NMC; **Table 1**), SNP genotyped using the Axiom Japonica V2 array, served as an independent validation set. Imputation accuracies for *LILR* CNV in this set closely matched those of the internal validation test results: *LILRB3* achieved 100% accuracy, while *LILRA3* and *LILR*A6 achieved 98.1% and 96.7% accuracy, respectively.

**Table 1:** Summary of prediction accuracies (call rate) based on LILR Japanese model as reference for independent Japanese validation set model as reference on independent Japanese validation set.

	LILR genes		
	LILRB3	LILRA6	LILRA3
Japonica array^®^ v2			
No. of SNPs	49	49	43
No. of training samples	419	419	363
No. of validation samples	190	180	188
No. of missing SNPs (%)	1 (2%)	1 (2%)	0 (0%)
Accuracies % (call rates %)	100.0 (100)	96.7 (100)	98.1 (100)

Furthermore, using our internal validation results, we predicted the most likely miscalled CNVs before quality control in the Japanese dataset (**Supplementary Table 1**). For example, miscalled *LILRA6* CN0 and CN2 were most commonly mistaken for *LILRA6* CN1. Conversely, miscalled *LILRA6* CN1 was frequently referred to as *LILRA6* CN2.

### 1kGP *LILR* CNV imputation accuracies

**Figure 4:**
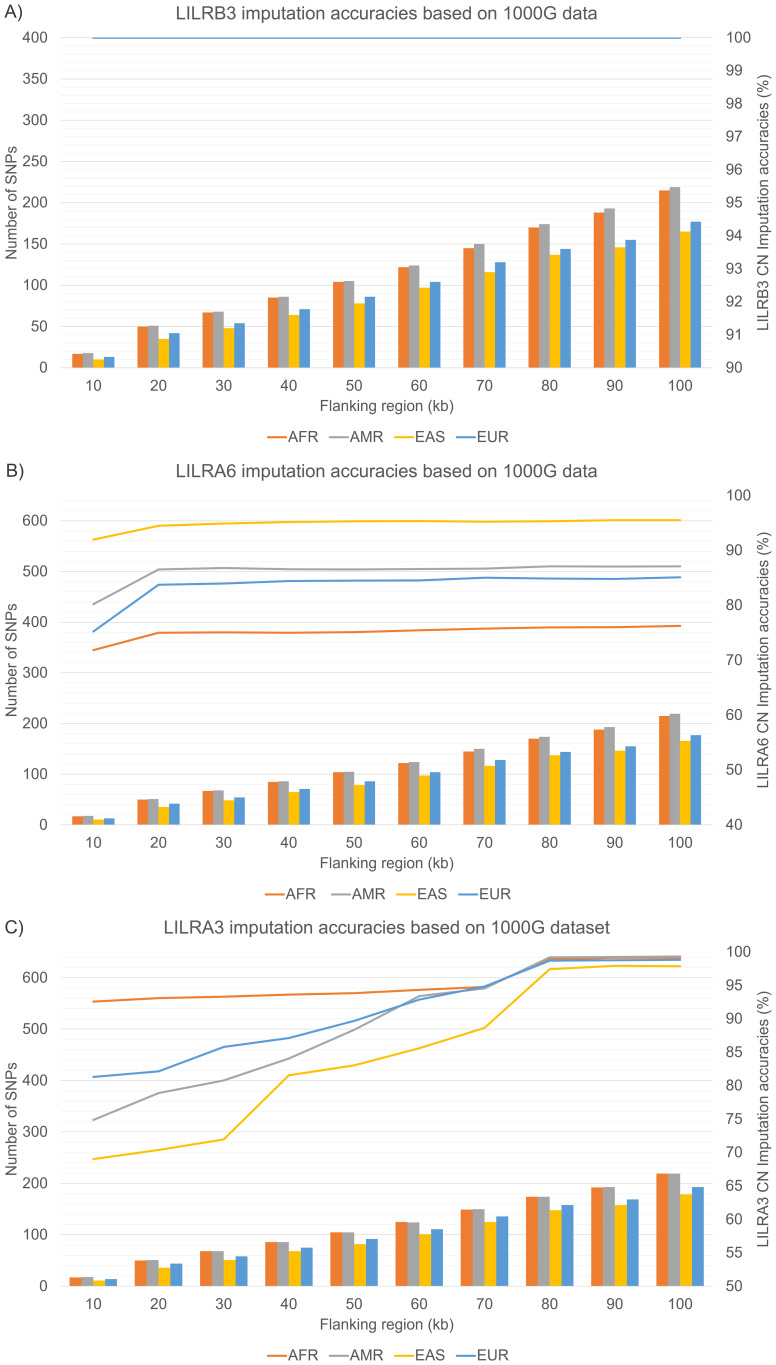
Internal validation of *LILR*s CN imputation accuracies across four 1000 Genome Project superpopulations. **(A)** *LILRB3* **(B)** *LILRA6* **(C)** *LILRA3*. AFR, African; AMR, Admixed American; EAS, East Asian; EUR, European; CN, Copy Number.

All 1kGP samples were genotyped using the Illumina Omni2.5 microarray. A minor discrepancy was observed in the number of SNPs available within the 10-100kb flanking region across different populations. For instance, in the case of *LILRA6* (**Figure 4B**), the 1kGP-EAS population exhibited the fewest SNPs, ranging from 10–165, which were utilized for the *LILR* CNV imputation model. This was followed by the 1kGP-EUR with SNPs ranging from 13–177, the 1kGP-AFR with 17–215 SNPs, and finally, the 1kGP-AMR with 18–219 SNPs. A similar trend was observed for *LILRB3* (**Figure 4A**) and *LILRA3* (**Figure 4C**).

In our internal validation test (**Figures 4A–C**), *LILRB3* achieved 100% accuracy, attributable to the lack of CNV in the dataset. In contrast, *LILRA6*’s CNV imputation reached peak accuracies of 94.5% (1kGP-EAS), 86.6% (1kGP-AMR), 83.8% (1kGP-EUR), and 75.0% (1kGP-AFR), particularly beyond the 20 kb flanking region. Similarly, *LILRA3* CNV imputation accuracy gradually increased, peaking at the 80 kb flanking region: with 1kGP-AMR reaching 99.2% and 98.9% for 1kGP-AFR, 98.7% for 1kGP-EUR, and 97.5% for 1kGP-EAS.

Moreover, we forecast the most probable CNV misidentifications across all miscalls before quality control in the 1kGP dataset (**Supplementary Table 2**). Our analysis miscalled *LILRA6* (0CN, 2CN, or 3CN) was most often identified as *LILRA6* 1CN. Similarly, *LILR*A3 1CN is most likely to be misclassified as 0CN, and vice versa.

### Call threshold evaluation

Call thresholds, reflecting confidence in *LILR* CNV imputation quality, were established for each imputed CNV pair. The optimal cutoff for CT was determined by internal validation, considering CNV imputation success rates (call rates). Overly stringent CT can diminish call rates, while overly lenient CTs would fail to adequately exclude low-quality imputed CNVs.

**Figure 5:**
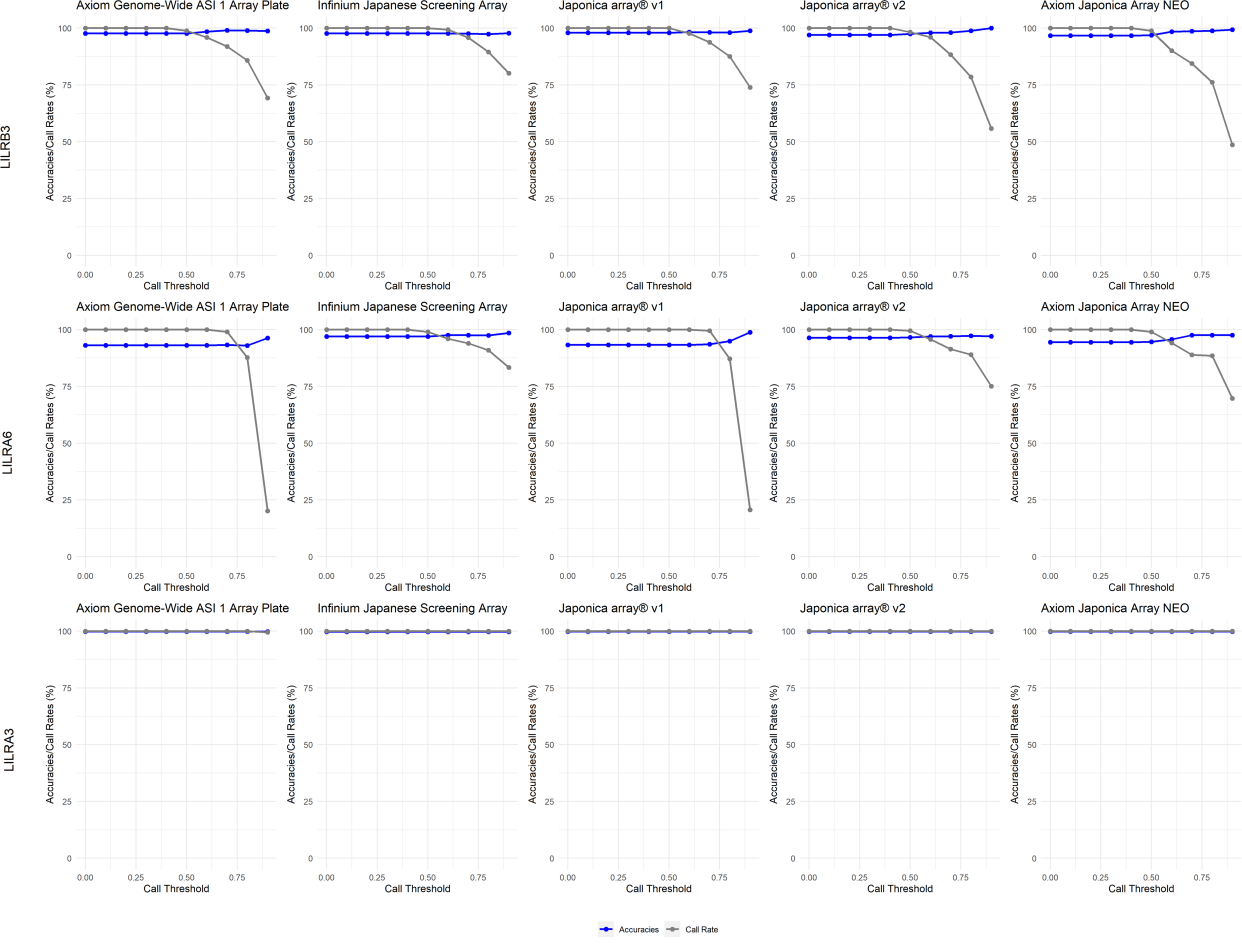
Call threshold evaluations of *LILRB3*, *LILRA6* and *LILRA3* on five Asian/Japanese-specific SNP genotyping arrays: Axiom Genome-Wide ASI 1 Array Plate, Infinium Japanese Screening Array, Japonica array^®^v1, Japonica array^®^v2 and Axiom Japonica Array NEO.

In the Japanese dataset (**Figure 5**), a 0.5 CT effectively filtered low-quality imputed CNVs, as shown by the accuracy results, while improving sample call rates. This trend was observed across all five SNP genotyping arrays examined in this study. Conversely, a CT exceeding 0.75 significantly reduces call rates and is therefore not recommended. For LILRB3 (**Supplementary Table 3**), among the 5 SNP genotyping arrays, the sensitivity (SEN) ranged from 89.9% to 100%. The specificity (SPE) also ranged from 89.9% to 100%. The Positive Predictive Value (PPV) ranged from 95.4% to 99.8%, while the Negative Predictive Value (NPV) ranged from 95.4% to 99.8%. Subsequently, for LILRA6 (**Supplementary Table 4**), among the 5 SNP genotyping arrays, the SEN varied from 26.3% to 100.0% (notably low SEN for the Affymetrix Axiom™ Japonica Array™ NEO for LILRA6 2CN at 26.3%). The SPE ranged from 18.5% to 100% (notably low SPE for the Affymetrix Axiom™ Japonica Array™ NEO for LILRA6 1CN at 18.5%). The PPV varied from 88.2% to 100%, and the NPV ranged from 90.5% to 100%. Lastly, for LILRA3 (**Supplementary Table 5**), among the 5 SNP genotyping arrays, the SEN varied from 89.9% to 99.3%, the SPE varied from 89.9% to 99.3%, the PPV varied from 95.4% to 98.9%, and the NPV varied from 95.4% to 98.9%.

**Figure 6:**
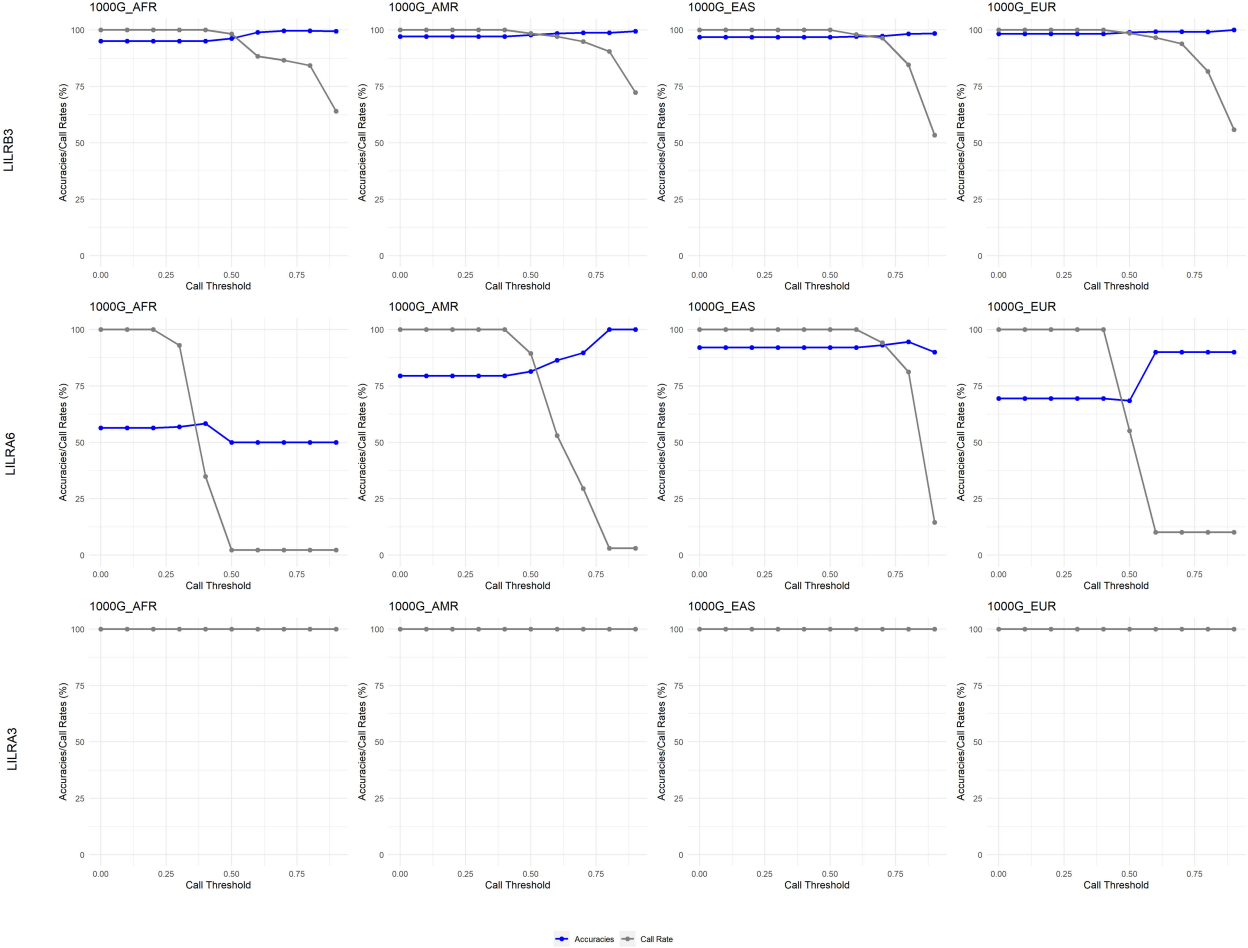
Call threshold evaluations of *LILRB3*, *LILRA6* and *LILRA3* across four 1000 Genome Project superpopulations. AFR, African; AMR, Admixed American; EAS, East Asian; EUR, European; CN, Copy Number.

Generally, a 0.5 CT is also suitable for the 1kGP dataset (**Figure 6**), except for 1kGP-EUR for *LILR*A6, where 0.4 is essential to maximize the call rate.

## Discussions

This study introduced LIBAG, a tool designed for CNV imputation, exclusively using SNP genotype data from SNP microarray datasets. The current version focuses on three *LILR* genes with known substantial CNV: *LILRB3*, *LILRA6*, and *LILRA3*. CNV in the LRC region complicates the design of distinct SNPs on conventional SNP genotyping arrays that adequately account for CNV in this area. Therefore, instead of relying on SNPs within the *LILR* genes, which exhibit high CN, LIBAG utilizes SNPs in their flanking regions of *LILRB3*, *LILRA6*, and *LILRA3*. This approach mitigates the issue of inadequate SNPs in *LILR* regions and facilitates CNV imputation for these genes.

In this study, we successfully developed a population-specific *LILR* CN imputation system and validated it internally and externally. Using a Japanese dataset genotyped on five distinct SNP microarray platforms, we directly assessed *LILR* CN imputation efficiency (**Figure 2**). SNP density surrounding the *LILR* and *LILR* CN distribution were directly linked to *LILR* CN accuracy. *LILRA6* CN accuracies reached up to 98.0% with the Infinium Japanese Screening Array, while *LILRA3* and *LILRB3* CN accuracies remained stable at approximately 99%. External validation using an independent NMC dataset corroborated the interval validation results, achieving 96.7% accuracy for *LILRA6* and 98.1% for *LILRA3*. This finding highlights the robustness of the *LILR*-CN imputation panel. Our findings indicate that population-specific SNP microarrays, such as the Infinium Japanese Screening Array and Axiom Japonica arrays, are more efficient at capturing population-specific CNV, as demonstrated by the CN distribution across various populations (**Figures 2**, **3**).

LILR receptors interact with conserved HLA motif epitopes located within the α3 domain and/or the highly conserved β2 microglobulin structure. Inhibitory LILRB receptors, recognize HLA class I molecules, particularly those expressing non-classical HLA-G (20). These interactions trigger inhibitory signals, suppressing the immune response mediated by HLA class I proteins, thereby promoting immune tolerance (21).

Genome-wide association studies (GWAS) have revealed that several *LILR*s are associated with diseases, such as Takayasu arteritis (*LILRB3/LILRA3*) (22, 23) and prostate cancer (*LILRA3*) (24). The effect of CNV on these conditions requires further investigation; however, a positive correlation exists between the relative expression levels of LILRA6/LILRB3 mRNA and the CNV genotype, with higher copies of LILRA6 resulting in an elevated LILRA6/LILRB3 ratio (25). Therefore, higher LILRA6 copy numbers may induce a shift towards an activation phenotype. Additional functional and large-scale population-based case-control studies will offer insights into the functional significance of CNV for these *LILR*s. Case-control analysis of disease should be accompanied by an examination of classical HLA class I molecules, including HLA-A, -B, and -C, as well as non-classical HLA-G, and their interactions with LILRs.

## Data Availability

Publicly available short-read whole-genome sequencing (srWGS) data were downloaded from the International Genome Sample Resource database (https://www.internationalgenome.org/data-portal/data-collection/30x-grch38). The SNP microarray dataset, genotyped using the Illumina Omni2.5 microarray (Illumina, CA, US), was downloaded from http://ftp.1000genomes.ebi.ac.uk/vol1/ftp/release/20130502/supporting/hd_genotype_chip/ .
